# The Report on the Draft Poor-Law Order

**Published:** 1913-09-20

**Authors:** A. C. Gibson

**Affiliations:** Late Matron, Birmingham Poor-Law Infirmary.


### THE REPORT ON THE DRAFT POOR-LAW ORDER.

### The Needs of the Sick in the Smaller Rural Workhouses.

By A. C. GIBSON, Late Matron, Birmingham Poor-Law Infirmary.

The long expected Report on the "Draft Poor-Law Institutions Order" has at last appeared, and makes in most ways sorry reading. It is neither possible, nor do we wish here to deal with any parts of this remarkable document, except so far as they have to do with the care of the sick. We have no interest in the relative position or importance of any "officer" except in so far as that position or importance affects the proper and (one would have thought) necessary care of, and attendance on, those who are suffering. It is remarkable that the Departmental Committee seem to have failed entirely to recognise that the representations made to them, on a large and difficult subject (on which none of them were experts), only in a very minor degree, and only in so far as that degree affected the sick, had anything at all to do with the importance, or otherwise, of the various ranks and positions of officials.

The whole point of the discussion as to the nursing in workhouses resolves itself into the question, Are the sick who have the misfortune to come to these institutions to be nursed, or are they not? We gather from the new Order that such persons as are lucky enough to be inmates of a workhouse containing 100 sick have the right to be cared for by qualified and competent nurses, but those who are innocently condemned to another containing, say, twenty-five sick inmates, may be relegated to the tender mercies of an unskilled attendant. Now, it has to be remembered that numbers have no connection with the painfulness or severity of illness; it is not only possible, but distinctly probable, that in an institution containing this charmed number there are no more cases requiring skilled and tender care than there are in one containing a fourth of the number. There can be no question that the nursing of the sick inmates of rural workhouses is surrounded by difficulty, but it seems a feeble and futile plan to decide that the difficulty is not to be faced and overcome, and simply to allow the existing archaic and cruel conditions to continue. Yet it is not easy not to give up any effort at the amelioration of the sufferings of the sick poor when we hear that, "after obtaining the views of the Poor-Law medical inspectors and of the lady inspectors, the Committee feel that it is not practicable to require the appointment of a fully trained nurse in every Poor-Law institution."

Much improvement in the conditions existing in the rural workhouses had been hoped for and expected from the appointment of trained lady inspectors, and it reduces the persons interested (alas! far too few) in the unfortunate inmates of such institutions almost to despair to know that a trained nurse inspector can be induced to believe that, where there are people who require a competent nurse, they must do without her, because it is "impracticable" to get her. One wonders if the deadening influences of a public department have already overcome the natural instincts of some of these ladies, and taken away from them much of their professional enthusiasm and sympathy. This paragraph goes on to express a pious opinion that, "even if she could be found, such a position (the tending and care of the helpless and the hopeless) would not be good for her." This seems a quite unnecessary interpolation; the Order is not concerned with the good of the nurse, except as it affects the good of the sick poor. If she is in good health, and keeps so, and is also good at her work, and kind to her patients, it need have no further concern on her account—beyond the necessary (and unexercised) care that she be fairly treated and allowed to carry on her work without any lay interference. It may safely and strongly be affirmed that the remedy advocated by one Association—namely, "the appointment of a fully trained nurse in every institution"—is the obvious and only cure for present evils, and that the cure is not "impracticable," and only requires some wisdom of organisation, and some patience in carrying out reforms.

Patience is only usefully exercised when some great object has to be attained, and it takes a great deal of that greatly over-estimated quality to enable anyone to go on with this most humane and righteous effort to lessen the burdens of the sick who are under the chilling care of the Poor Law. It is quite safe to say that there is nothing impracticable in the suggestion—so callously put on one side. All that is needed is to make the conditions of service such that a self-respecting, well-trained woman can accept them without danger of deterioration in her ideals and actions. It is no question of precedence, or of authority; it is the question of the right of every trained woman to do the special work she has given years to make herself proficient in without the interference (except in actual institutional discipline) of untrained supervision. And, further—and of far greater importance—it is the right of every sick person to have all the skilled care and the comfort possible to alleviate suffering or restore health. When Poor-Law nurses are enabled to feel that they can do their duty to their patients in every particular, and can carry out those niceties of conduct and skill which they have been taught to think right and proper; when their work is free from the worry and wear of lack of linen and dressings and utensils, and so on, they will be forthcoming, and will do their work heartily and honestly. But for years the positions of even the nurses under the Poor Law have been most difficult, and in many cases impossible; many of the few really trained women who have tried to give ease and help to the inmates of the small Unions have found their lives intolerable, and given up in despair. This being so, it will be long before the dislike of accepting the old conditions passes away, and some time may probably elapse before any considerable number of really competent women are available. If it were known that irksome regulations were withdrawn, and that in every small Union the nurse would be permitted to do her work unmolested, the positions in even the smallest Unions would gradually be filled. Also it must be remembered that for fifty years the position of nurses in Poor-Law institutions has been anomalous, and has consequently become deservedly most unpopular, and the Local Government Board would doubtless have to practise that patience, which they have so relentlessly taught others to practise, before all small Unions had their complete staff. But it would come.

There are many excellent nurses, well trained, efficient in every detail of nursing experience, to whom the small number of sick would be a real joy, women who only want a quiet sphere, and have no hankering after excitement, or popularity, or kudos. There are many equally capable and equally desirable, who are not strong enough for the continuous rush of hospital, or the irregularity of private work, and who would be entirely suitable for, and entirely satisfied with, such a life. No amount of money wasted on advertising, and no other inducements of any kind, will attract those women while the conditions of work remain unchanged. It is almost incomprehensible that this fact has not been grasped by the nursing and medical inspectors of the Local Government Board.

When those conditions are altered, if they ever are, the young nurses now being trained in the large infirmaries will become available for those positions, which, by their training, they are so competent to fill, but which any conscientious professional adviser must, at present, advise them to avoid. It is quite possible for every Union, however small, in course of time, to have a fully trained nurse who will not deteriorate, and so cause anxiety to the Departmental Committee; but there is only one way of doing this, and if the statement of "impracticability" is accepted we must, so far as in us lies (and few of us will find it easy), resign ourselves to the fact that many of our poor brothers and sisters are suffering unnecessary discomfort and pain from a not easily but certainly removable cause.

The able-bodied, the aged, the children have at least some one to turn to; but the sick, who most of all need it, have no one.

### The Personnel of the Committee.

It is, perhaps, ungracious to refer to the Committee, who were none of them experts on nursing matters, and who doubtless took much trouble, and acted according to their light. This Committee consisted of four gentlemen—one of them a clerk to the Guardians of a large Union, the others Local Government Board officials. None of them would seem to have had any special knowledge of the needs of the sick in small Unions. Yet the Order, or most of it, appeared in September in the *Poor Law Officers' Journal* before any nursing body had, so far as is known, been consulted. When its great inadequacy and injustice was recognised, two Associations, one consisting entirely of trained nurses (the Poor-Law Matrons' Association), and the other of ladies, partly nurses and partly lay members, who had given years of work, and thought, and help to the nursing of rural workhouses (Workhouse Nursing Association), asked for leave to send deputations to state their views. They were allowed this privilege, and on separate days each sent a selection of their members to the Local Government Board Offices. The latter Association dealt entirely with the question of the rural workhouses; the former, quite naturally, with the questions affecting the superintendent nurses. Both were most courteously received and attentively listened to, and this, of course, was better than not being heard at all. But no advice or information was invited by those gentlemen; in both cases the interview was asked for, and had it not been asked for the Order might have been even worse than it is. What possible chance could there be for big questions needing much experience and long consideration to be thrashed out and considered in two interviews of less than an hour, giving no chance of reply to adverse statements, or discussion of objections?

### A "Must" of the Future.

If we are to have further Orders issued, efforts must be made to have representatives on the Committee who thoroughly understand the whole question of both large and small workhouse sick wards, for in this way only can any advance in the care of the sick be brought about. In no other way can any Order dealing with the question of the nursing of the sick, which so sorely needs reform, be satisfactorily dealt with and brought up to modern requirements and demands.

Space will not allow of even a reference to the alterations in the position of superintendent nurse, though on that point and others much just criticism might be offered. The outcome of it all is, first, that there is nothing impracticable in the suggestion that every small Union must have at least one fully trained nurse; secondly, that conditions of service must be made more possible; and when this happens nurses will be forthcoming.

For above thirty years this question of the nursing of the sick in rural workhouses has been very near to the hearts of many. Money and brains, and time and strength, have been willingly and ungrudgingly spent in trying to alleviate existing conditions. The case of the sick has been laid before the authorities over and over again with so little result that it seems almost hopeless to dream that "Good may be the final end of ill." Like the Queen of Sheba, too many of those who have worked hard to remedy this glaring injustice have "no spirit left," and are wondering if it is any use struggling on. But such workers are not likely to settle a difficulty by finding it "impracticable," and it would seem that all that is left is to hope and work for the hastening of the day when every sick person will be given the attention he or she deserves at the hands of another Government Department.

